# Supplementary material for: MetaRibo-Seq measures translation in microbiomes
Source: Nat Commun. 2020 Jun 29;11:3268. doi: 10.1038/s41467-020-17081-z (PMC7324362; doi:10.1038/s41467-020-17081-z)
Supplement: Supplementary file 10 — Supplementary Data 7 [file 41467_2020_17081_MOESM10_ESM.zip › File2/Confidence_High_Taxonomy/369440_out.krona.html]

Javascript must be enabled to view this page.

members
magnitude
magnitudeUnassigned
count
unassigned
taxon
rank

369440\_out

5

2
superkingdom
5

1239
phylum
5

5
class
186801

order
186802
5

5
family
186803

genus
2569097
5

5
species
39488

SRS015431\_contig\_number\_54647SRS018427\_contig\_number\_contig-100\_111.142445SRS019068\_contig\_number\_24857SRS144183\_contig\_number\_33989SRS148159\_contig\_number\_30782
